# Granzyme A Stimulates pDCs to Promote Adaptive Immunity via Induction of Type I IFN

**DOI:** 10.3389/fimmu.2019.01450

**Published:** 2019-06-26

**Authors:** Kanako Shimizu, Satoru Yamasaki, Maki Sakurai, Noriko Yumoto, Mariko Ikeda, Chiemi Mishima-Tsumagari, Mutsuko Kukimoto-Niino, Takashi Watanabe, Masami Kawamura, Mikako Shirouzu, Shin-ichiro Fujii

**Affiliations:** ^1^Laboratory for Immunotherapy, RIKEN Center for Integrative Medical Sciences, Yokohama, Japan; ^2^Laboratory for Protein Functional and Structural Biology, RIKEN Center for Biosystems Dynamics Research, Yokohama, Japan; ^3^Laboratory for Integrative Genomics, RIKEN Center for Integrative Medical Sciences, Yokohama, Japan

**Keywords:** granzyme A, dendritic cell, TLR9, type I IFN, innate immunity, adaptive immunity, adjuvant, anti-tumor effect

## Abstract

Granzyme A (GzmA), together with perforin, are well-known for their cytotoxic activity against tumor or virus-infected cells. In addition to this cytotoxic function, GzmA stimulates several immune cell types and induces inflammation in the absence of perforin, however, its effect on the dendritic cell (DC) is unknown. In the current study, we showed that recombinant GzmA induced the phenotypic maturation of plasmacytoid DCs (pDCs) and conventional DCs (cDCs), but not their apoptosis. Particularly, GzmA made pDCs more functional, thus leading to production of type I interferon (IFN) via the TLR9-MyD88 pathway. We also demonstrated that GzmA binds TLR9 and co-localizes with it in endosomes. When co-administered with antigen, GzmA acted as a powerful adjuvant for eliciting antigen-specific cytotoxic CD8^+^ T lymphocytes (CTLs) that protected mice from tumor challenge. The induction of CTL was completely abolished in XCR1^+^ DC-depleted mice, whereas it was reduced to less than half in pDC-depleted or IFN-α/β receptor knockout mice. Thus, CTL cross-priming was dependent on XCR1^+^cDC and also type I IFN, which was produced by GzmA-activated pDCs. These results indicate that GzmA -stimulated pDCs enhance the cross-priming activity of cDCs *in situ*. We also showed that the adjuvant effect of GzmA is superior to CpG-ODN and LPS. Our findings highlight the ability of GzmA to bridge innate and adaptive immune responses via pDC help and suggest that GzmA may be useful as a vaccine adjuvant.

## Introduction

Among the many attempts to develop new and improved vaccine strategies for infectious and cancer, a major obstacle is the lack of appropriate adjuvants that can safely drive potent cellular immunity (1). The classical adjuvants, aluminum salts (alum) and incomplete Freund's adjuvant (IFA), which are used clinically, promote humoral immunity and T helper type 2 (Th2) cell responses, but their ability to efficiently drive T helper 1 (Th1) responses and CTLs is limited (2). However, recent reports have described DMXAA and cGAMP, adjuvant ligands for the cGAS-STING pathway, that can promote type I IFN production, resulting in induction of cellular immunity (3–5). Understanding of adjuvanticity and its mechanisms is critical for the rational design of new and improved vaccines. Since dendritic cells (DCs) have a superior ability to present antigen to naive T cells, understanding of how DCs can be augmented by adjuvants is essential (6).

DCs comprise the heterogeneous subsets of conventional DCs (cDCs) and plasmacytoid DCs (pDCs) and can orchestrate the type of innate and adaptive immune response (7–9). In evaluating the activity of adjuvants, especially for the induction of T cell immunity, it is extremely useful to assess the *in situ* maturation of cDCs by adjuvants using several criteria (10–12); (1) high expression of MHC class I and II, (2) up-regulation of costimulatory molecules, including CD80 and CD86 and (3) production of inflammatory cytokines such as IL-12, or chemokines (13–16). (4) Another important factor is cross-presentation by DCs, i.e., whether T cell immunity *in vivo* occurs after co-administration of antigen plus adjuvant (17–19).

Antigen priming of T cells *in vivo* occurs by fine-tuning the interaction between lymphocytes and DCs within secondary lymphoid organs (13–16). Besides the interaction between peptide antigen-bearing DCs and T cells, pDCs have often been shown to contribute to optimal cell-mediated responses (20). pDCs are known for their ability to produce large amounts of type I interferon (IFN) in response to viral infection or stimulation by toll-like receptor-7 (TLR7) and TLR9-ligands (20). There are also some reports of pDC-cDC cross-talk in generating immune responses (21–23).

Type I IFNs are widely recognized for their roles in antiviral immunity (24). They consist of 16 members in mice, 12 IFN-α subtypes, IFN-β, IFN-ϵ, IFN-α, and IFN-υ. Type I IFN by itself stimulates not only effector T cells, but also DCs, promoting their maturation and survival and the enhancement of DC cross-presentation (25–32). Indeed, the co-injection of a high dose of soluble IFN-α/β plus antigen (25), or the injection of DC-targeted DEC205-binding antigen in combination with the IFN-α/β inducer poly(I:C) (26) stimulates T-cell priming. Small amounts of endogenous type I IFN acting on DCs have also been reported to be essential for CD8^+^T cell priming in tumor models (27).

Granzymes are a family of homologous serine proteases involved in inducing apoptosis in virus-infected cells and tumor cells in the presence of perforin (33). Effective cytotoxic lymphocytes, including CD8^+^ cytotoxic T lymphocytes (CTLs), NK cells, γδT cells and NKT cells, contain granzyme A (GzmA) and granzyme B (GzmB) that are packaged into granules(33–35). In the immunological synapse formed between tumor cells and effector cells, effector cells (CTL or activated NK cells) expressing GzmB have high cytotoxic activity against tumor cells, but those expressing GzmA) do not (36–39).

There is emerging evidence that granzymes have other non-cytotoxic activities that do not require perforin. Granzyme M was shown to be an important mediator for the release of MIP-1α from NK cells during early microbial infection and also to play a critical role in mediating the early stages of gut mucosal inflammation (40, 41). Granzyme K was shown to bind to LPS and augment LPS-CD14 complex formation, thereby enhancing monocyte activation (42). GzmA promotes proinflammatory cytokine production including in the context of infectious diseases and rheumatoid arthritis (33, 36, 43, 44). In fact, various proinflammatory cytokines, such as IL-1β, TNF-α, IL-6, and IL-8 are induced by GzmA stimulation from LPS pre-sensitized macrophages in a TLR4-dependent manner or from monocytes treated with TLR2/TLR4 ligands (43–45). However, the effect of GzmA on DCs remains to be determined.

Here, we report the potential of GzmA to affect DC maturation. Our studies indicate that GzmA serves as a potent adjuvant capable of driving DC-mediated antitumor cellular immunity.

## Methods

### Mice and Cell Lines

SPF 6-8 wk old C57BL/6 were purchased from CLEA Japan. TLR2-KO, TLR3-KO, TLR4-KO, TLR7-KO, TLR9-KO, MyD88-KO, and TRIF-KO mice were purchased from Oriental Bioservice or obtained from Dr. Akira (Osaka Univ.). Batf3-KO and BDCA2-diphteria toxin receptor (DTR) mice were purchased from Jackson Laboratories. IFNαβR-KO and XCR1-DTR-venus mice were provided by Dr. T. Kaisho (Wakayama Univ.). TAP-KO mice were provided by Dr. RM. Steinman (The Rockefeller Univ., New York, USA). CD11c-DTR-GFP mice were provided by Dr. D. Littman (NYU, New York, USA). OT-I TCR transgenic (Tg) mice (B6 background) were originally provided by Dr. WR. Heath (Walter and Eliza Hall Institute, Victoria, Australia) and Ly5.1 congenic OT-1 mice were generated by cross/backcross breeding of OT-1 and C57BL/6-Ly5.1 mice. All the mice were maintained under specific pathogen-free conditions and all procedures were performed in compliance with the protocols approved by the Institutional Animal Care Committee at RIKEN. The HEK293T cell line was obtained from the RIKEN CELL BANK. B16 melanoma and MO4 (OVA-expressing B16 melanoma) cell lines were described previously (46).

### Reagents

LPS and CpG-ODN were purchased from Invivogen. DNase I and RNase were purchased from Ambion and Invitrogen, respectively. α-GalCer was purchased from Funakoshi. Mouse Flt3L were purchased from Peprotech. Anti-Flag-tag (SIGMA), anti-cMyc-tag (Clontech), anti-TLR9 (Santa Cruz) and anti-GzmA (rabbit polyclonal) (LSbio) antibodies were from the indicated suppliers. Anti-CD11c (HL3), -CD40 (3/23), -CD80 (6-10A1) and -IFN-γ were purchased from BD. Anti-CD8 (53.67), -CD45.1 (A20), -CD86 (GL-1), -Siglec H(551) and Annexin-V-APC were purchased from Biolegend. Anti-B220 (RA3-6B2) was purchased from e-Bioscience. OVA-tetramers were purchased from MBL. CFSE and Mito-tracker were purchased from Molecular Probes. OVA_257−264_ peptide (SIINFEKL) was obtained from Toray Research Center, Inc. Chloropromazine and Latrunculin A were purchased from Wako Co. Diphtheria toxin were purchased from Sigma.

### Recombinant GzmA Production and Purification

The gene encoding residues 29-260 of mouse GzmA was cloned into the baculovirus transfer vector pBAC-3 (Novagen) as a fusion with the gp64 signal sequence at its N-terminus and a histidine tag at its C-terminus. High-titer recombinant baculoviruses were obtained using the flashBAC GOLD Baculovirus Expression System (Oxford Expression Technologies). *Spodoptera frugiperda* cells derived from superSf9-1 (Oxford Expression Technologies), were cultured in Sf-900™III SFM (Invitrogen, Thermo Fisher Scientific Corp.), infected with the viral stock at a multiplicity of infection of 0.1 and incubated with shaking at 27°C for 72 h. The culture supernatants were concentrated using the QuixStand System (GE healthcare) and then applied to a HisTrapHP column (GE healthcare) equilibrated with a buffer containing 50 mM imidazole, 20 mM Tris-HCl (pH 8.0), and 1 M NaCl. The target protein was eluted with a 50-500 mM linear imidazole gradient in 20 mM Tris-HCl (pH8.0), 1M NaCl and diluted 2-fold with 2xPBS buffer. The GzmA was further purified using a Bio-Scale™ Mini CHT™ Type I ceramic hydroxyapatite cartridge (Bio-Rad) equilibrated with 2xPBS buffer and was eluted with an NaCl (0-1M linear gradient) in 3x PBS. Purified GzmA was diluted 3- fold with distilled water and concentrated by ultrafiltration with an Amicon Ultra-15 30K-MWCO filter (Millipore). GzmA batches were not contaminated with endotoxin (<0.01 EU/ml) as determined using a ToxinSensor Chromogenic LAL Endotoxin Assay (GeneScript), which utilizes a modified Limulus Amebocyte Lysate to detect endotoxin.

### Cell Preparation

Mononuclear cells from the spleen and BM were isolated and DCs were prepared from spleen as previously described (47).

### Generation of Bone Marrow DC Subsets

The tibias and femurs were removed from mice and flushed to obtain bone marrow cells. After lysing red cells, bone marrow mononuclear cells were cultured with complete medium (RPMI-1640 containing 10% FCS, 2 mM l-glutamine, 100 U/ml penicillin, 100 μg/ml streptomycin, 50 μM 2-ME, 100 μM nonessential amino acids and 1 mM sodium pyruvate) in the presence of murine Flt3L (200 ng/ml) (Peprotech) for 8–10 days. Then, bone marrow-derived DCs (FL-DC) were harvested by vigorous pipetting and removal of non-adherent cells. In order to sort pDCs/ cDCs from total DCs, the cells were stained with anti-mouse CD11c(HL3)-PE/Cy7 and B220(RA3-6B2)-FITC and Aqua fluorescent reactive dye from the LIVE/DEAD Fixable dead cell kit (Molecular Probe).

### Analysis of DC Maturation

FL-DC (2 × 10^5^ cells/well) were stimulated with LPS (100 ng/ml) or GzmA (1 μg/ml) for 24 h. The cytokine levels in culture supernatants were measured using ELISA Kits for IFN-α, IFN-β (PBL) and IL12p40 (BD). The cells were also analyzed for phenotypic maturation. In some experiments, GzmA (0.5 μg) was pre-treated with 2 unit DNase I (Ambion) or 100 μg/ml RNase A (Invitrogen) at 37° C for 30 min, or was heat inactivated at 70° C for 30 min. pDCs (B220^+^CD11c^dim^) (1 × 10^5^ cells/well) or cDCs (B220^−^CD11c^+^) (1 × 10^5^ cells/well) sorted from FL-DCs were also stimulation with LPS (100 ng/ml), CpG (1 μg/ml) or GzmA (1 μg/ml) for 24 h.

### RNA-Seq Analysis

Sorted pDC and cDC (2 × 10^5^ cells/well) were stimulated with 1 μg/ml GzmA for 8 h and then total RNA was extracted using TRIZOL reagent (Ambion). RNA-seq libraries were prepared using the NEBNext Ultra RNA Library Prep Kit for Illumina (New England Biolabs) according to the manufacturer's protocols and were sequenced on a HiSeq1500 DNA sequencer (Illumina single read, 50 bp). The sequence reads were mapped to the mouse genome (NCBI version 37) using TopHat2 (version 2.0.8) and botwie2 (version 2.1.0) with default parameters and gene annotation was provided by the NCBI RefSeq. The transcript abundances were estimated using Cufflinks (version 2.1.1). Cufflinks was run with the same reference annotation as TopHat2 to generate FPKM (fragments per kilobase per million mapped reads) values for known genes.

### Flow Cytometry

Cells were preincubated with anti-CD16/32 Ab to block Fcγ receptors, then washed and incubated with the indicated mAbs for 30 min. Cells were washed and analyzed on a FACS Calibur or a Canto II FACS cytometer (BD). Cytokine expression by CD8^+^ T cells was determined using a protocol for intracellular cytokine staining (48). Briefly, splenic cells were incubated in the presence of Golgi Plug (BD Bioscience) for 6 h with or without OVA peptide_257−264_ and then incubated with antibodies to surface markers. The cells were then permeabilized in Cytofix-Cytoperm Plus (BD Biosciences) and stained with an IFN-γ mAb. For analysis of apoptosis, cells were stained with Annexin-V-APC-APC (Biolegend) and Propidium Iodide (PI) (Sigma-Aldrich) and then assessed by flow cytometry.

### Plasmids

#### Myc-GzmA

Total RNA was extracted from mouse naïve lung cells. The coding sequence of the *gzma* gene was once amplified by reverse transcription-PCR using a OneStep RT-PCR Kit (Qiagen) and cloned into the *Hin*dIII and *Eco*RI sites of pGEM-4Z (Promega). To obtain the coding sequence of the processed form of gzma, the part of the sequence encoding Ile29-Val260 was re-amplified by PCR using a forward primer designed to attach an ATG initiation codon to the 5′ end of the template and was cloned into the *Xho*I site of pCMV-Myc-C (Clontech) to generate a carboxy-terminally Myc-tagged expression construct. Deletion variants (exons 2-3, 3-4, and 4-5) were constructed from the above plasmid by PCR mutagenesis using PrimeSTAR Mutagenesis Basal Kit (TaKaRa). For pCMV-Myc-gzmA (exons 2-3) (Ilu29-Arg118), the forward primer 5′- TTGTACGGGAGCAGAAGCTGATCTCA−3′ and reverse primer 5′- TCTGCTCCCGTACAAGTTGTAGATC−3′ were used. For pCMV-Myc-gzmA (exons 4-5) (Leu119-Val260), the forward primer 5′-CCACCATGCTAAAGAAAAAAGCAACA−3′ and reverse primer 5′-TCTTTAGCATGGTGGCTCGAGAGAT−3′ were used. pCMV-Myc-gzmA(exon 3-4) (Gly73-Asn208) was constructed as follows: first, exon 5(Gly209-Val260) was deleted from processed form of full length of gzmA(exon 2-5, Ile29-Val260) using a forward primer (5′- CCTGCAATGAGCAGAAGCTGATCTCA-3′) and a reverse primer (5′- TCTGCTCATTGCAGGAGTCCTTTCC-3′) and next, exon2 (Ilu29-Val72) was deleted using a forward primer (5′- CCACCATGGGAAAGAGATCTAAGTTC-3′) and a reverse primer (5′-TCTTTCCCATGGTGGCTCGAGAGAT-3′). Cloned DNAs were all verified by sequencing.

#### 3xFLAG-TLR9

Mouse TLR9 cDNA was purchased from InvivoGen. The coding sequence was amplified by PCR and cloned into the *Xho*I site of pCMV-3Tag-8 (Agilent) using In-Fusion HD Cloning Kit (Clontech) to generate a carboxy-terminally FLAG-tagged expression construct.

### Immunoprecipitation Assays

HEK293T cells were co-transfected with mTLR9-3xFlag and gzmA-cMyc using lipofectamine 2000. Twenty-four hours later, cells were harvested and whole cell lysates were used for immunoprecipitation. In brief, cells were lysed with lysis buffer (20 mmol/L Tris HCl, pH 7.4, 150 mM NaCl, 1% NP40) (2 × 10^7^ cells/mL). The lysates were incubated at 4°C with rotation for 30 min and then cleared by centrifugation at 16,000 × g for 20 min. To pre-clear, 20 μL of Protein G Sepharose (GE healthcare) or Dynabeads™ Protein G (Invitrogen) were added and incubated for 20 min at 4°C under gentle rotation. Then, the lysates were incubated with 50 μL of Protein G Sepharose or Dynabeads™ Protein G, and 1 μg of anti-FLAG monoclonal antibody (M2, Sigma) for 4–12 h at 4°C under gentle rotation. Immunoprecipitates were washed three times with 1 ml of the lysis buffer or wash buffer (PBS containing 0.01% Tween 20). Proteins were eluted with Laemmli's SDS loading buffer, separated on a 4-20% gradient SDS-polyacrylamide gel (BIO-RAD), and electro-transferred to a PVDF membrane (Immobilon-P, Millipore). The membranes were blocked with TBST (20 mM TrisHCl pH 7.4, 150 mM NaCl, and 0.05% Tween20) containing 1% skim milk and incubated with the primary antibodies: monoclonal antibodies (anti-FLAG M2 or anti-Myc 4A6) at a 1:1000 dilution for 4 h at room temperature or overnight at 4°C. The membranes were washed three times with TBST for 10 min and incubated at room temperature with HRP-conjugated goat anti-mouse IgG antibodies (R&D) or TrueBlot®:anti-mouse IgG (Rockland) at 1:1000 for 1 h. The membranes were then washed with TBST three times and the immune complexes were visualized using a chemiluminescence system and detected with LAS1000 (Fuji film). To compare the binding activity to TLR9 between full length GzmA and deletion mutants, the amount of immunoprecipitated GzmA proteins was normalized to the input level and then the binding activity of mutants was calculated relative to full-length GzmA. In an immunoprecipitation assay to detect the direct binding interaction of GzmA with endogenous TLR9 in primary FL-DCs, we treated FL-DCs with 3 μg/ml of GzmA and incubated them for 8 h. FL-DCs were lysed with lysis buffer and the cell lysates were precleared with ProteinG Sepharose as described above. Then the lysates were incubated with 50 μL of Protein G Sepharose and 1 μg of anti-TLR9 monoclonal antibody (5G5, Santa Cruz) for 4–12 h at 4°C under gentle rotation. After several washings with lysis buffer, proteins were boiled and separated on SDS-PAGE and subsequently probed with anti-TLR9 antibody or anti-GzmA antibody.

### Confocal Microscopy

Recombinant GzmA was labeled with Alexa488 using an Alexa488 protein labeling kit (Molecular Probes). To evaluate the GzmA-TLR9 interaction, Alexa488-GzmA was added to sorted pDCs cultures and then cells were harvested at the indicated time points. The cells were washed and then permeabilized with Cytofix-Cytoperm Plus (BD Biosciences) and blocked with 10% goat serum (SIGMA) in Perm/Wash buffer (PW) (BD) for 15 min at RT. Anti-Mouse TLR9-Ab (1:100, BD) and anti-mouse Rab7 Ab (1:100, Abcam) were used as primary antibodies. The cells were incubated with the antibody solution for 30 min at RT. After washing with PW, cells were stained with secondary antibodies, goat anti-mouse IgG Cy3 (1:500, Jackson ImmunoResarch) (1:500) and goat anti-rabbit IgG Alexa647 (1:500, Invitrogen) in PW for 30 min at RT. Nuclei were labeled by staining with DAPI. Subsequently, cells were washed and cytospun onto a slide and then mounted with Fluorescence Mounting Medium (DAKO). For the analyses of the localization of GzmA, Alexa488-GzmA was added to sorted pDCs and then the cells were harvested at the indicated time points. The cells were then stained with Mito-Tracker Deep Red (Molecular Probes) and DAPI. Immunofluorescence images were obtained using a confocal laser scanning microscope (TCS SP2 and TCS SP5; Leica) and images were transferred to Adobe Photoshop 7.0. Intensity line profiles of a region of interest were analyzed with quantify mode of LAS AF software (Leica). A minimum of 10-20 cells were examined per slide. The calculation method for (%) colocalization is as follows: (%) Colocalization = (The number of puncta positive for all of GzmA, TLR9 and Rab7)/(The number of puncta positive for any of GzmA, TLR9 and Rab7), or (The number of puncta positive for both GzmA and mitochondria)/(The number of puncta positive for either GzmA or mitochondria). Mean (%) colocalization = Average of (%) colocalization.

### Assays for Adjuvant Activity of GzmA

To assess the role of GzmA as an adjuvant for a T cell-mediated immune response, we used OVA as antigen. OVA in association with dying osmotically shocked, syngeneic, TAP-KO splenocytes (T/O) can be useful as a model of a cell-associated antigen. To prepare this cell-associated OVA antigen expressing cells (T/O), spleen cells from TAP-KO mice were incubated with hypertonic medium at 37°C in the presence of 10 mg/ml OVA (SEIKAGAKU Corp.) for 10 min, and further incubated with hypotonic medium for 2 min to induce apoptosis, followed by washing with cold PBS. To evaluate the dose of OVA in T/O cells, cell lysates were measured using an OVA ELISA kit (ITEA Inc.) To examine the adjuvant activity, mice were immunized with T/O cells (2 × 10^7^ cells/mouse) with or without GzmA (20 μg/mouse), LPS (20 μg/mouse), CpG-ODN (30 μg/mouse), or α-GalCer (2 μg/mouse) at day 0. A week later, these immunized mice were tested for T cell priming by quantifying the frequency of OVA-tetramer^+^ CD8^+^ T cells and OVA-specific IFN-γ producing cells in the spleen. In some experiments, BDCA2-DTR mice as recipients were immunized with T/O plus GzmA and treated with diphtheria toxin (DT) (10 ng/g) i.p. daily from day-2 to day 0 and then every other day until day 7. T cell responses were assessed 7 days later.

### Antigen Presentation Assay

For the depletion of DC subsets (pDC, cDC and XCR1^+^DC subsets) *in vivo*, BDCA2-DTR mice, CD11c-DTR mice and XCR1-DTR-venus mice were treated with DT (10 ng/g for BDCA2-DTR, 4 ng/g for CD11c-DTR and 25 ng/g for XCR1-DTR-venus mice) i.p. at day−2,-1, 0, and +1. Ly5.1^+^OT-1 cells were labeled with 5 μM CFSE (Invitrogen) in PBS for 5 min at 37 ° C and then unincorporated dye was quenched by adding FBS. Cells were washed three times with PBS. CFSE-labeled CD8^+^Ly5.1^+^OT-1 cells (1 × 10^6^ cells/mouse) were transferred into C57BL/6 or DT-treated BDCA2-DTR, CD11c-DTR and XCR1-DTR-venus mice. On the following day, the mice were given i.v. injections of 1 × 10^7^ T/O cells together with GzmA (20 μg/mouse). CFSE dilution of OT-1 splenic T cells was analyzed 3 days later after gating on CD8^+^Ly5.1OT-1 T cells.

### Tumor Experiments

C57BL/6 mice were immunized with T/O cells together with GzmA as described above. Two wk later, the mice were challenged with 1 × 10^5^ MO4 or B16 melanoma cells s.c. Tumor growth was monitored by measuring three perpendicular diameters. Tumor volume was calculated according to the formula V = L x W^2^ × 0.52, where V is the volume, L is the length, and W is the width.

### Ethics Statements

All procedures were performed in compliance with the protocols approved by the Institutional Animal Care Committee at RIKEN.

### Statistical Analysis

The *p*-values were calculated using the paired-Student's *t*-test, the Mann-Whitney U test, Kruskall-Wallis test, Steel's many*-*one rank sum test or one-way ANOVA test, as appropriate. *P* < *0.05* was considered statistically significant.

## Results

### GzmA Induces the Maturation of cDCs and pDCs

**Figure 1:**
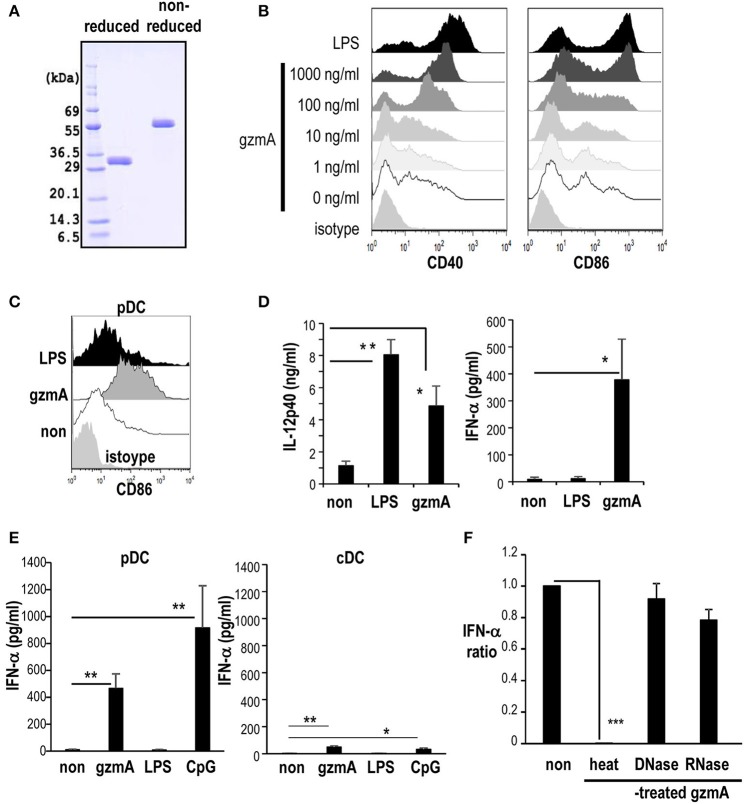
GzmA promotes DC maturation. **(A)** Recombinant GzmA is predominantly dimeric. Reduced and non-reduced GzmA were run on 10–12 % SDS-PAGE and stained with Coomassie Brilliant Blue. **(B)** Phenotypic maturation. FL-DCs were generated from BMMNCs from C57BL/6 mice in the presence of Flt3L. The expression of costimulatory molecules (CD40 and CD86) on FL-DCs (CD11c^+^ cells) were analyzed 24 h after stimulation with graded dose of GzmA or LPS (100 ng/ml) (*n* = 3). **(C)** Expression of CD86 on the pDC (CD11c^dim^B220^+^) of FL-DCs after stimulation with GzmA (1 μg/ml), LPS (100 ng/ml) (*n* = 5). **(D)** Cytokine production by FL-DCs stimulated with GzmA. FL-DCs were cultured in the presence of GzmA or LPS for 24 h and the supernatants were assayed for IL-12p40 and IFN-α by ELISA (mean ± SEM, *n* = 5). ^*^p < *0.05*, ^**^p < *0.01* (Kruskal Wallis test). **(E)** IFN-α production by pDCs and cDCs. pDCs, and cDCs were sorted from FL-DCs and stimulated with GzmA (1 μg/ml), LPS (100 ng/ml), or CpG-ODN (1 μg/ml) for 24 h and the supernatants were assayed for IFN-α by ELISA (mean ± SEM, *n* = 4–6). ^*^p < *0.05*, ^**^p < *0.01* (Kruskal Wallis test). **(F)** Effect of treatment of GzmA with nucleases or heat. GzmA was pre-treated with nothing (non), DNase, RNase or heat-inactivated and then added to FL-DCs. Supernatants from the cultures were analyzed the production for IFN-α. The data show the ratio of IFN-α production in the GzmA treated:untreated cultures, the latter set to 1 (mean ± SEM, *n* = 5). ^***^p <*0.001* (Kruskal Wallis test).

To begin to address the effect of GzmA on DCs, we first produced recombinant GzmA protein for use of the current study. We verified that it was predominantly dimeric under non-reduced conditions (Figure 1A). GzmA batches were not contaminated with endotoxin (endotoxin level <0.01 EU/ml) as determined by LAL assay. Some reports indicated that GzmA synthesized using *E. coli* systems is contaminated the endotoxin, i.e., LPS (45). LPS is a known stimulator for DCs and has been used as an adjuvant in some vaccines. Therefore, we used the GzmA synthesized using a non-bacterial baculovirus method and used LPS as a positive control DC stimulator in the current study. The effect of GzmA on FLT3L-driven BM-DCs (FL-DCs), which include both conventional DCs and pDCs, was tested *in vitro*. GzmA upregulated the CD40 and CD86 maturation markers of FL-DCs (Figure 1B). There was a dose-dependent increase in the phenotypic maturation and, at a dose of 1 μg/ml, their expression was similar to the level achieved by treatment with LPS (Figure 1B). Then, the DC subpopulations in FL-DCs were evaluated. GzmA phenotypically matured both pDCs and cDCs (Figure 1C and Figure S1A). In terms of cytokine production, GzmA-stimulated FL-DCs produced IFN-α, but LPS-stimulated FL-DCs did not (Figure 1D, right). On the other hand, LPS-stimulated FL-DCs produced more IL-12 than GzmA-stimulated FL-DCs. (Figure 1D, left). In fact, after sorting, we found that GzmA-stimulated pDCs produced IFN-α (Figure 1E), but little IFN-β (IFN-α 466.1 ± 107.2 pg/ml vs. IFN-β 44.1 ± 28.7 pg/ml). In contrast, cDCs produced less IFN-α than pDCs in response to GzmA (Figure 1E) and very little IFN-β (IFN-α 49.0±9.3 pg/ml vs. IFN-β 8.3 ± 5.0 pg/ml). LPS-stimulated cDCs produce IL-12 relatively higher than GzmA-stimulated cDCs (Figure S1B). Therefore, in this study, we focused on the effect of GzmA on pDCs. In terms of pDCs capable of producing IFN-α, we used CpG-ODN as a positive control. When we evaluated IFN-α production by comparing GzmA with CpG-ODN, CpG-ODN-stimulated pDCs produced more IFN-α than GzmA-stimulated cells *in vitro* (Figure 1E). So, we determined to compare the adjuvant effect of these three reagents in *in vivo* studies later. Next, we excluded the possibility that the response was due to DNA or RNA contamination of the GzmA. For this purpose, we treated GzmA with nucleases prior to the culture. DNase- or RNase treatment of GzmA did not affect IFN-α production by pDCs, whereas heat-denatured GzmA protein could no longer activate pDCs (Figure 1F).

### GzmA Enters DCs via Endocytosis

**Figure 2:**
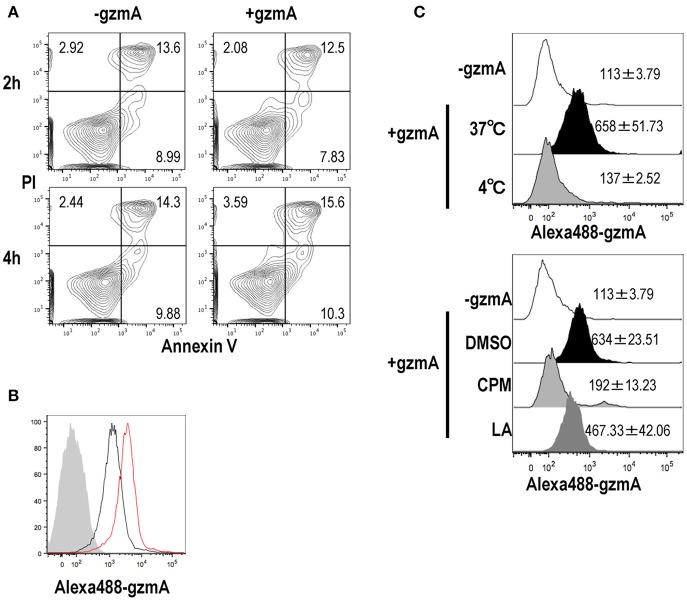
GzmA is endocytosed by pDCs but does not induce apoptosis. **(A)** Assessment of cell viability and apoptosis in response to GzmA. After FL-DCs were cultured in the presence of GzmA for 2 (upper) or 4 (lower) h, they were stained with Annexin-V and PI (*n* = 3). **(B)** Uptake of GzmA by pDCs. Alexa488-labeled GzmA were added to FL-DC cultures. The uptake of GzmA by pDCs (CD11c^dim^B220^+^) was analyzed by flow cytometry: before (shaded), 2 (black), and 4 (red) h later (*n* = 3). **(C)** Same as in **(B)**, but FL-DCs were cultured at 4°C or 37°C during pulsing with GzmA for 2 h (upper). Endocytosed of GzmA by pDCs was assessed by FACS analysis. In other experiments (lower), pDCs were pre-treated with DMSO, Chloropromazine (CPM) (20 μg/ml) and Latrunculin A (LA) (5 μM) for 40 min before adding Alexa488-GzmA. Then, endocytosed of GzmA by pDCs was assessed by FACS 2 h later. Numerical data indicate the MFI of Alexa488-GzmA (mean ± SEM, *n* = 3).

GzmA released from CTL can diffuse across the plasma membrane into the cytosol of target cells through perforin pores in killing target cells. However, it is unknown how GzmA can be taken up by pDCs. We suspected that DCs would undergo apoptosis after internalizing GzmA, however when we assessed apoptosis of FL-DCs, there was no difference in the GzmA -treated and -untreated cells (Figure 2A). We then assessed the uptake of GzmA by FL-DCs. Alexa-488-labeled GzmA was added to DC cultures and could be detected in pDCs 2 h later, with an increase in staining intensity at 4 h (Figure 2B). To rule out the possibility that GzmA was not being actively internalized but was just binding to the cell membrane, we cultured FL-DCs in the presence of GzmA at different temperatures. GzmA was taken up at 37°C but not at 4°C (Figure 2C, upper), indicating that the uptake occurred via an energy-dependent process. The endocytic pathways can be classified into several types: phagocytosis, micropinocytosis and endocytosis. The mechanism of GzmA uptake by pDCs was evaluated using specific inhibitors of these pathways. Latrunculin A (LatA), a specific phagocytosis and macropinocytosis inhibitor, partly reduced uptake of GzmA (Figure 2C, lower). Chlorpromazine (CPM), an inhibitor of endocytosis, significantly reduced the uptake of GzmA, suggesting that uptake mainly occurs via endocytosis (Figure 2C). Thus, we confirmed that pDCs mainly uptake GzmA by endocytosis and that this uptake does not lead to apoptosis.

### Stimulation of the TLR9-MyD88 Pathway and Gene Transcription of GzmA-Matured DCs

**Figure 3:**
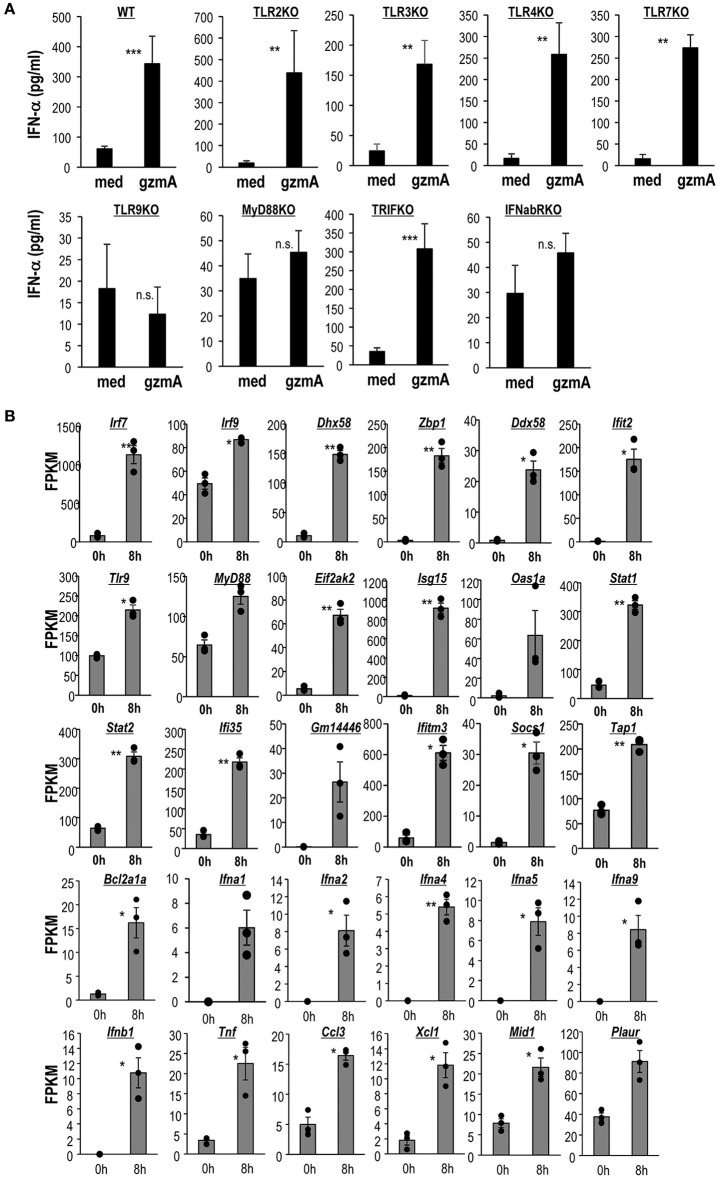
Activation of the TLR9-MyD88 signaling pathway and a distinct transcriptomic signature of GzmA-stimulated pDCs. **(A)** IFN-α production by FL-DCs from WT or several types of KO mice stimulated with GzmA. FL-DCs were cultured in the presence of GzmA for 24 h and the supernatants were measured by ELISA for IFN-α (mean ± SEM, *n* = 4) medium vs. GzmA, ^**^p < *0.01*, ^***^p < *0.001* (Mann-Whitney). **(B)** Analysis of differential gene expression in pDCs before and after stimulation with GzmA. pDCs unstimulated or stimulated with GzmA for 8 h were sorted. Differential genes associated with IRF signaling, TLR signaling, IFN signaling, MyD88 signaling, and interferon-responsive genes in pDCs were assessed by RNA-Seq. The gene expression analysis was performed using an R based package, EdgeR with the raw counts from RNA-seq data. Then, expression levels (expressed as FPKM) from selected genes of pDCs under these two conditions were compared (*n* = 3, mean ± SEM). ^*^p < 0.05, ^**^p < 0.01 (paired Student's *t*-test).

It is well-known that CpG-ODN, but not LPS stimulates pDCs in a TLR9-dependent manner (49, 50). Therefore, the involvement of TLR pathways was assessed by analyzing eight strains of knock-out (KO) mice. We found severely reduced IFN-α production and a decrease in expression of CD86 in GzmA-stimulated FL-DCs derived from TLR9-KO and MyD88-KO mice, but not from other TLR-KO mice or TRIF-KO mice (Figure 3A and Figure S2). In addition, pDCs derived from IFNα/βR-KO also failed to respond to GzmA (Figure 3A). These findings indicate that TLR9 and MyD88 pathways are required for the GzmA signal and that IFN-α signaling in an autocrine pathway is involved in DC activation.

We further studied the enhanced signals related to TLR9 and MyD88 in pDCs by RNA deep sequencing. It is well-known that TLR9 activates IRF7 pathway via MyD88 to induce type I IFN in pDCs (49). To assess the enhancement of signaling in GzmA-stimulated pDCs, we sorted pDCs and stimulated them with GzmA, harvested them 8 h later and then performed transcriptome analysis (Figure 3B). Exposure of pDCs to GzmA resulted in substantial augmentation of IRF signaling (*Irf7, 9, Zbp1, Dhx58, Ddx58*, and *Ifit2*), TLR signaling (*Tlr9, Myd88*, and *Eif2ak2*), MyD88 signaling *(Bcl2a1a, Ifna1,Ifna2, Ifna4, Ifna 5, Ifna9, Ifnb1, Tnf, Ccl3, Xcl1, Mid1*, and *Plaur)* and interferon-responsive genes (*Isg15, Isg20, Oas1,2, Stat1,2, Ifi35, Gm14446, Ifitm3, Socs1*, and *Tap1*) (Figure 3B). We thus verified that the TLR9- MyD88-IRF7-type I IFN pathways are enhanced when pDCs are activated by GzmA.

### Association of GzmA and TLR9

**Figure 4:**
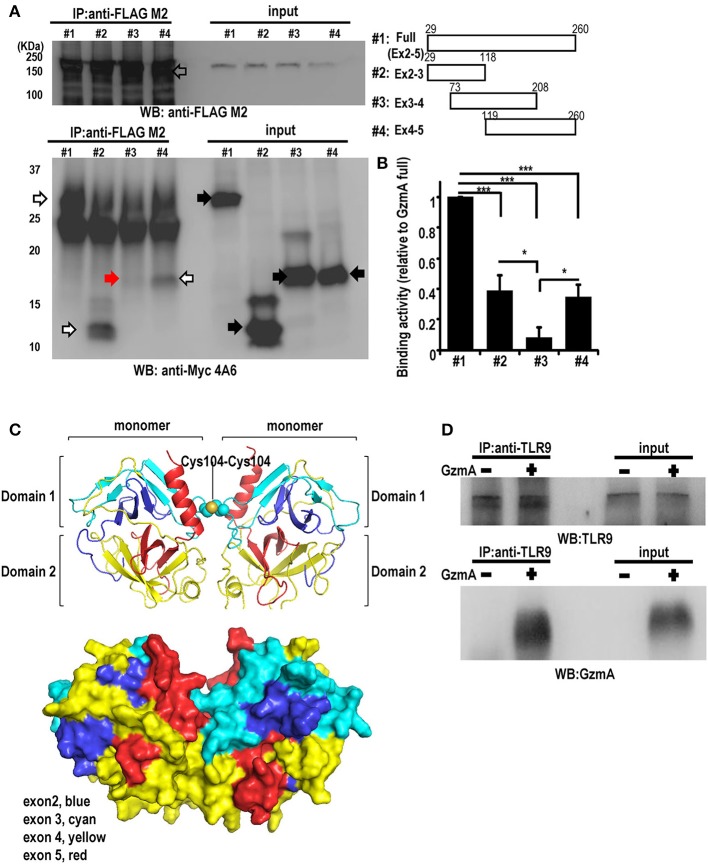
Interaction between GzmA and TLR9. **(A)** Co-immunoprecipitation western blot (WB) assay for the interaction between GzmA and TLR9. cMyc-tagged full-length or truncated GzmA proteins (right) and flag-tagged TLR9 protein were expressed in HEK293T cells (left). Whole-cell lysates were subjected to immunoprecipitation using anti-Flag Ab and immunoblotted with anti-cMyc Ab. **(B)** Quantification of binding activity was assessed by the ratio of IP-GzmA (white and red arrows) (lower left): the ratio of input GzmA (black arrow) was calculated from that of full-length GzmA (mean ± SEM, *n* = 4). ^*^p < *0.05*; ^***^ *p* < *0.001* (one-way ANOVA test with Tukey's *post-hoc* test). **(C)** 3D structural model of mouse GzmA dimer (upper panel) generated using SWISS-MODEL Repository with its surface representation in the same view (lower panel) (72, 73). GzmA monomers are colored by exon (exon2, blue; exon 3, cyan; exon 4, yellow; exon 4, red). The Cys104-Cys104 disulfide bond between monomers is shown by spheres. This figure was created with the program PyMOL. **(D)** Co-immunoprecipitation western blot (WB) assay for the interaction between GzmA and TLR9 in primary FL-DCs. Whole-cell lysates were harvested 8 h after culture in the absence (1) or presence (2) of GzmA. These were immunoprecipitated using anti-TLR9 and immunoblotted with anti-TLR9 or anti-GzmA Ab. Results are representative of three independent experiments.

TLR9 is reported to bind damage-associated molecular patterns (DAMP) proteins; the direct association of TLR9 with HMGB1 was previously shown in HEK293 cell models (51). Therefore, to determine if there was direct binding of GzmA to TLR9, we transfected HEK293T cells with mouse TLR9-Flag and GzmA-cMyc and performed co-immunoprecipitation (IP) studies. The results showed clear interaction of TLR9 and GzmA (Figure 4A). Deletion analyses showed that protein domains encoded by exon 2-3 and by exon 4-5 co-immunoprecipitated TLR9 (white arrow), but a fragment encoded by exon 3-4 failed to do so (red arrow) (Figures 4A,B). These findings indicate that two distinct regions, encoded by exon 2 and exon 5, are involved in the interaction of GzmA with TLR9. Based on the crystal structure of human GzmA (52), we generated a homology model of mouse GzmA used in this study (Figure 4C). Based on this structure, exon 2-3 and exon 4-5 correspond to domain 1 and domain 2 of the trypsin-like region of the GzmA monomer, respectively. Some loop regions (exon 2 and exon 5) and the C-terminal helix (exon 5) are located on the same side of the molecular surface. Thus, it is possible that some of these regions may participate in the binding with TLR9. To confirm that the TLR9-GzmA interaction was not an overexpression artifact, we also examined the direct binding interaction of GzmA to endogenous TLR9 in primary FL-DCs, including pDCs, by co-immunoprecipitation studies (Figure 4D). Total cell lysate of pDC that had been treated with or without GzmA were immunoprecipitated with anti-TLR9 antibody. As shown in Figure 4D, co-immunoprecipitation of endogenous TLR9 and GzmA was observed only in GzmA-treated pDC. This finding confirmed the interaction of GzmA and TLR9 in a physiological setting.

### Co-localization and Interaction Between GzmA and TLR9 in pDC

**Figure 5:**
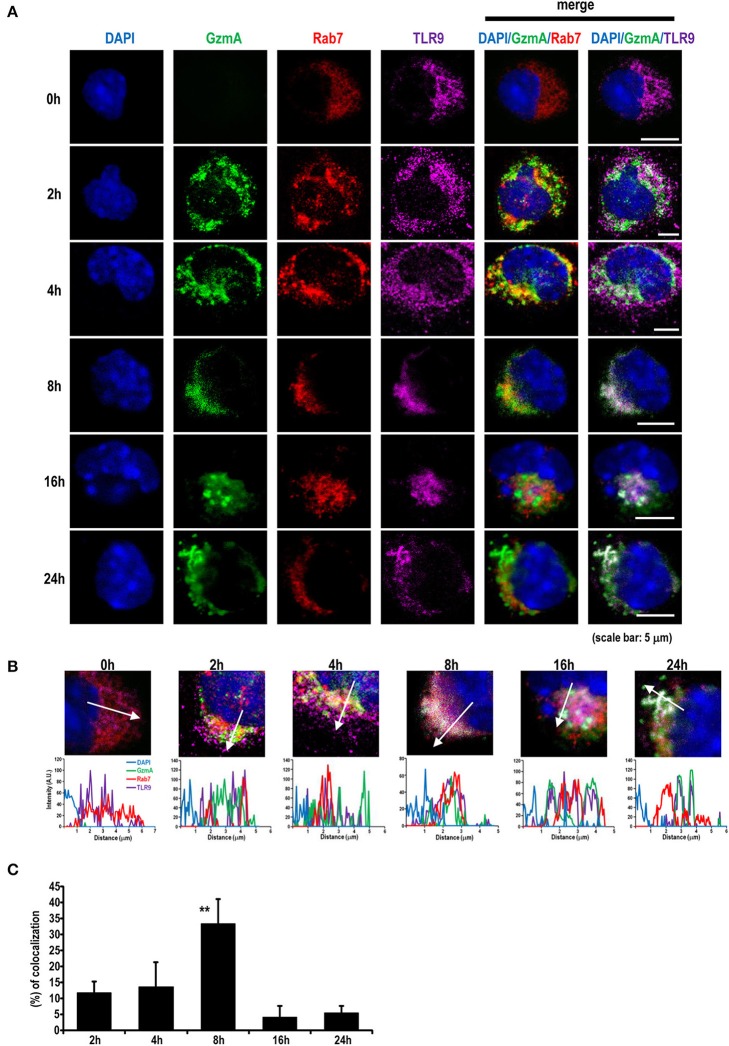
GzmA colocalizes with TLR9 in the late endosome compartment. **(A)** Representative images showing localization of Alexa488-labeled GzmA (green) in pDCs. Sorted pDCs incubated with Alexa488-labeled GzmA (green) were harvested at the indicated time points after GzmA treatment and stained with anti-TLR9 (magenta) and anti-Rab7 (red) antibodies and DAPI (blue). Scale bars, 5 μm. **(B,C)** Intensity line profiles from magnified images were assessed. Line scans represent variation in red, green, and magenta fluorescence intensity along the line denoted by an arrow and then the colocalization was quantified as described in the text (mean ± SEM, *n* = 5–9). ^**^p <0.01 (one-way ANOVA test with Tukey's *post-hoc* test).

**Figure 6:**
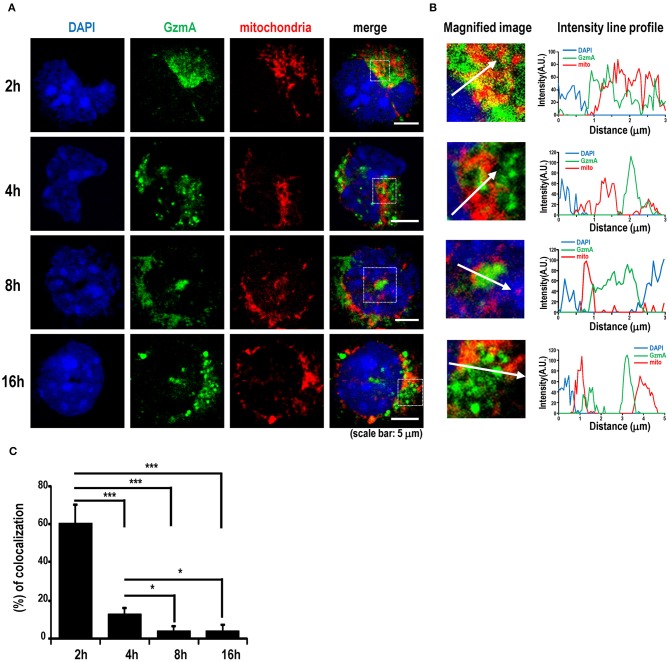
GzmA binds to mitochondria. **(A)** Representative images showing localization of GzmA in pDCs. Sorted pDCs incubated with Alexa488-labeled GzmA (green) were harvested at the indicated time points and stained with mito-tracker (red) to detect the co-localization of GzmA and mitochondria. Scale bars, 5 μm. **(B,C)** Intensity line profiles from magnified images were assessed. Line scans represent variation in red and green fluorescence intensity along the line denoted by an arrow and then the colocalization was quantified (mean ± SEM, *n* = 10). ^*^p < 0.05; ^***^ *p* < 0.001 (one-way ANOVA test with Tukey's *post-hoc* test).

Because it is known that TLR9 signaling can be activated in the endosome by viral DNA or exogenous CpG-DNA (49, 50), we next traced the localization of GzmA in pDCs. We assessed the intracellular co-localization of GzmA and TLR9 after adding Alexa488-labeled GzmA to pDCs (Figures 5A,B). The GzmA (green)-TLR9 (magenta) complex was detected by confocal microscopy in late endosomes (Rab7^+^) 4 h later, with a peak in co-localization at 8 h (Figure 5C). There was no co-localization of DAPI and GzmA, indicating that it does not enter the nucleus. We also found that GzmA (green) showed partial overlap with or in close proximity to the mitochondria (red) for just the first 2-4 h, but not at 8–16 h (Figures 6A–C). These findings suggested that an interaction of GzmA with endosome and/or mitochondria may play an important role in the stimulation of TLR9 signaling.

### GzmA Is an Immunoadjuvant for Anti-tumor Immunity

**Figure 7:**
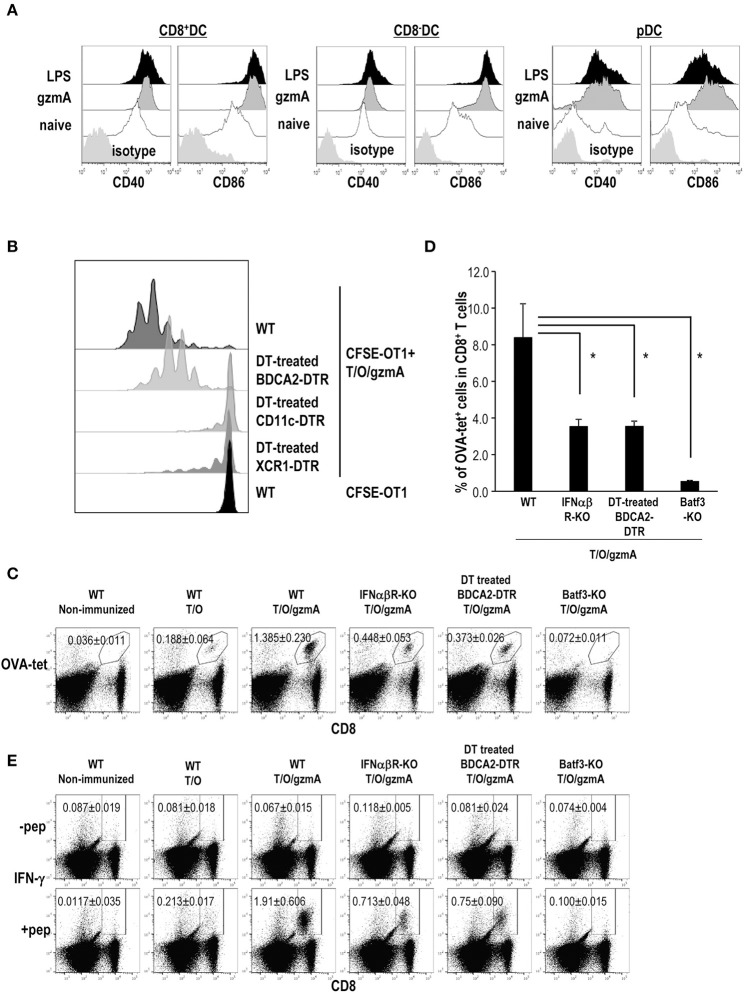
GzmA-stimulated pDCs promote XCR1^+^ DCs to cross-present antigen to CD8^+^ T cells. **(A)** *In vivo* DC maturation by GzmA. Mice were injected intravenously with GzmA (20 μg/mouse) or LPS (20 μg/mouse). Sixteen h later, splenic DC subsets (CD8^+^ or CD8^−^ cDCs and pDCs) were analyzed for expression of maturation markers CD40 and CD86 by flow cytometry. (*n* = 5) **(B)** Cross-presentation of antigen by DCs *in vivo*. BDCA2-DTR, CD11c-DTR and XCR1-DTR mice were treated with DT at day-2,−1, 0, and 1. Mice were administered with 1 × 10^6^ CFSE-labeled OT-1 cells at day-1 and then immunized with cell-associated OVA (T/O) together with GzmA at day 0. Three days later, OT-1 proliferation was monitored in spleen cells by CFSE dilution. Data are representative of two independent experiments (*n* = 3). **(C–E)** Antigen specific CD8^+^T cell response. As shown in **(B)**, but in this case the immune response was measured in naïve mice and several KO mice immunized i.v. with T/O cells together with or without GzmA. In some experiments, BDCA2-DTR mice were treated with DT. The frequency of CD8^+^T cells bearing a TCR specific for an OVA-H-2K^b^ tetramer **(C,D)** and IFN-γ production by OVA-specific CD8^+^ T cells **(E)** in spleen were analyzed by flow cytometry on day 7. All data in **(C–E)** are representative of two independent experiments with similar results (mean ± SEM, *n* = 4). ^*^ < *p* < *0.05* (WT vs. others, Steel's Many*-*one Rank Sum test).

We asked whether GzmA can act as an immunoadjuvant *in vivo*. First, we examined DC maturation *in situ* after intravenous injection of GzmA as compared to LPS. We examined cytokine production in serum and phenotypic changes of DCs *in situ* after intravenous injection of gzmA as compared to LPS. Administration of gzmA induced higher levels of serum IFN-α than LPS, but not IFN-β (Figure S3) and upregulation of costimulatory molecules on both CD8a^+^ and CD8a^−^ cDCs and pDCs in spleen (Figure 7A). We then tested the adjuvant activity of GzmA. For this study, cell-associated OVA antigen was generated by osmotic shock treatment of spleen cells from TAP-KO mice in the presence of OVA protein (hereafter, T/O). The T/O cells themselves cannot directly present antigen to T cells; they are dying cells that contain OVA protein (223.34 ± 32.76 ng/2 × 10^7^ cells). To monitor antigen presentation by this cell-associated vaccine form, we first adoptively transferred 1 × 10^6^ CFSE-labeled, OT-1 OVA-specific TCR transgenic T cells as reporter cells, and then injected 1 × 10^7^ T/O cells together with 20 μg GzmA. As a negative control, we detected weak T cell responses in mice injected with T/O alone (Figure S4A), as previously reported (17, 53). In fact, Liu et al. previously reported that vaccination with T/O led to T cell proliferation initially, followed by deletion of T cells (17). We detected no OT-1 proliferation in mice injected only with GzmA plus OT-1 cells (Figure S4B). We observed efficient OT-1 proliferation in WT type mice immunized with T/O plus GzmA (Figure 7B). To identify the DC subset involved in cross-presentation, we depleted total CD11c^+^DCs, the XCR1^+^DC subset or pDCs using transgenic mice, CD11c-DTR, XCR1-DTR-venus or BDCA2-DTR mice, respectively. Treatment of CD11c-DTR and XCR1-DTR-venus with diphtheria toxin (DT) totally ablated the presentation of OVA from the injected cell-associated OVA to OT-1 cells whereas OT-1 proliferation was only partially blocked in pDC-depleted mice (Figure 7B). These results indicate that cDCs, especially the XCR1^+^ DC subset, cross present cell-associated OVA antigen and that pDCs may not directly prime T cells but more likely supply XCR1^+^ DC with type I IFN to promote their maturation and cross-presentation according to previously published studies (31).

Next, we confirmed the antigen-specific CD8^+^ T cell response in WT type, IFNα/βR-KO, pDC-depleted and Batf3-KO mice without adoptive transfer of OT-1 cells. Since the treatment with DT for XCR1-DTR-venus mice is not possible for more than 1 wk (54), we used Batf3-KO mice as XCR1^+^ DC subset (i.e., CD8^+^DC in spleen and CD103^+^DC in non-lymphoid organs) deficient mice. The frequency of OVA-tet^+^ CD8^+^ T cells (Figures 7C,D) as well as antigen-specific IFN-γ production (Figure 7E and Figure S5) in IFNα/βR-KO and pDC-depleted mice was less than half of that in WT type mice, and was abolished in Batf3-KO mice. Taken together, our study suggests that GzmA can stimulate both cDCs and pDCs *in situ*, and that pDCs boost the induction of a strong and efficient T cell response through producing IFN-α which promotes antigen cross-presentation by XCR1^+^DCs.

**Figure 8:**
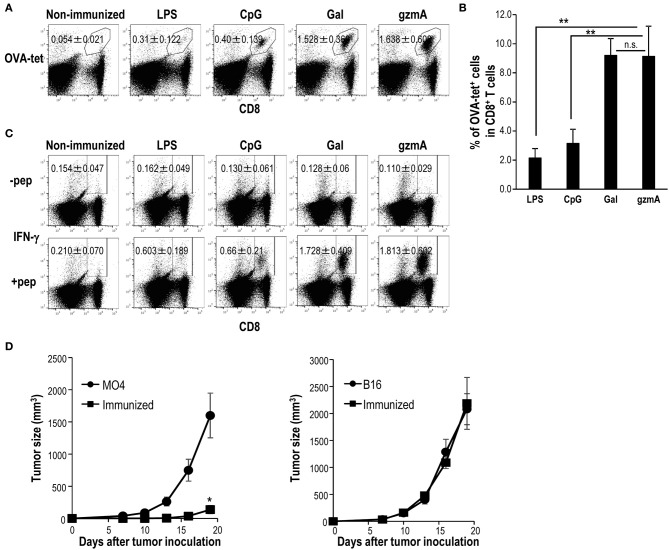
Adjuvant effect of GzmA on antitumor cellular immunity. **(A–C)** CD8 T cell response by different adjuvants. C57BL/6 mice were immunized intravenously with T/O together with LPS (20 μg/mouse), CpG-ODN (30 μg/mouse), α-GalCer (2 μg/mouse) or GzmA (20 μg/mouse). The frequency **(A,B)** and IFN-γ production **(C)** of OVA-specific CD8^+^ T cells in spleen were analyzed by flow cytometry on day 7. All data in **(A–C)** are representative of three independent experiments with similar results (mean ± SEM, *n* = 5) ^**^ < *p* < *0.01* **(**GzmA vs. others, Steel's Many*-*one Rank Sum test) **(D)** The anti-tumor adjuvant effect of GzmA. Mice were immunized with T/O cells together with GzmA (20 μg/mouse). One week later, mice were challenged with OVA-expressing B16 (MO4) or B16 (1 × 10^5^/mouse) melanoma cells, subcutaneously. The size of growing tumors was measured at the indicated time points (*n* = 5–6/group). ^*^p < *0.05* (Mann-Whitney).

To compare the adjuvant activity of GzmA, we used three different adjuvants known to stimulate DCs, LPS, CpG-ODN, and α-GalCer (NKT cell ligand). α-GalCer has been shown to be a superior adjuvant (55), and injection of α-GalCer can induce full maturation of DCs *in situ* via CD40/CD40L signaling and cytokines (IFN-γ and TNF-α), thus leading to adaptive immunity (18, 53). Surprisingly, antigen-specific T cell immunity driven by GzmA reached levels similar that induced by α-GalCer and was much higher than that induced by LPS or CpG-ODN (Figures 8A–C). In addition, mice vaccinated with T/O cell-associated OVA and GzmA were protected against the MO4 (OVA-expressing B16 melanoma) tumor (Figure 8D, left), but not against the parental B16 tumor (Figure 8D, right), demonstrating the antigen specific antitumor effect.

## Discussion

The rational development of successful vaccines requires an optimal adjuvant to promote the immune response, which depends on the status of antigen-presenting cells (1, 6). Particularly, a comprehensive understanding how adjuvants regulate DC-mediated adaptive immunity is crucial. In the current study, we found that GzmA acts as a powerful immunoadjuvant for eliciting antigen-specific CD8^+^ T cells. We elucidated the mechanism for this finding by following experiments. When vaccinated with GzmA plus antigen, vaccinated WT mice showed a strong T cell response, but DT-treated BDCA2-DTR, i.e., pDC-depleted mice or IFN-α/βR-KO mice immunized with GzmA had a reduced T cell response, thus indicating the importance of IFN-α-producing pDCs for the induction of activated T cells. On the other hand, vaccinated DT-treated CD11c-DTR or XCR1-DTR, i.e., CD11c^+^DCs, particularly XCR1^+^cDCs depleted mice did not show a T cell response. These results indicated that for induction of T cell immunity, the ability of GzmA to directly activate cDCs without the help of pDCs is weak, but critical, while the primary effect of GzmA via activation of pDCs plays an important role in full activation of killer T cells. Thus, GzmA potently activated pDCs for type I IFN secretion which in turn promoted CTL activation probably by boosting antigen cross-presentation by XCR1^±^ cDCs and their maturation. Accordingly, tumor antigen-specific vaccination with this adjuvant results in potent antitumor activity.

The immune system is triggered to respond to many exogenous pathogen-associated molecular patterns (PAMPs) via pathogen recognition receptors (PRRs) such as TLRs and RIG-I like receptors (49, 50). In addition, DAMPs, which include several antimicrobial peptides and proteins (AMPs) (e.g., LL37, defensin, cathelicidin, eosinophil-derived neurotoxin, granulysin), nuclear binding protein (HMGB1), heat shock proteins (HSP60 and 96), ion binding proteins (S100A8 and A9 and lactoferrin), and nucleotides/metabolites (ATP and uric acid), which are formed as result of cell death or tissue injury, can trigger the immune system. Thus, many structurally distinct multifunctional endogenous mediators can act as DAMPs (56, 57). Among DAMPs, alarmins are known to exert immune-stimulatory functions; however, not all of them function as adjuvants for the induction of cellular immunity. For example, LL37, HMGB1 and HSP 70, 90, and 96 activate macrophages and DCs though immune sensors and receptors, e.g., TLR2, 4, and 7-9 (51, 58–60). HMGB1 released from dying tumor cells stimulates TLR-2, 4 or 9 signaling in DCs leading to antitumor effects (61, 62). Also, HMGB1 enhances CpG-ODN-mediated TLR9-signaling, but it does not itself trigger type I IFN production (51). LL37, by forming a complex with extracellular DNA, activates pDCs capable of producing type I IFN, but *in vivo* administration of LL37 leads to tumor progression, despite the fact that both of these molecules can stimulate DCs (63, 64). To authenticate a molecule as a new adjuvant, it needs to be evaluated using *in vivo* studies to determine if it can be a useful vaccine adjuvant, particularly for recruitment and activation of immune cells, including DCs (49, 50). In the current study, our data suggest that GzmA may be a kind of alarmin based on both *in vivo* and *in vitro* studies.

All TLRs are type I transmembrane proteins and share a common architecture consisting of an ectodomain, a transmembrane region, and a cytosolic Toll-IL-1 receptor (TIR) domain that activates downstream signaling pathways (49). The function of each TLR has been stratified and characterized. TLRs are broadly classified into two categories depending on their cellular location (49, 65). TLR1, 2, 4, 5, 6, and 10 are located on the plasma membrane and these recognize lipids, lipoproteins and proteins. TLR3, 7, 8, 9, 11, 12 and 13 are located in the endosomal compartments and these recognize microbial or self-nucleic acids. Since some previous studies indicated that GzmA allows LPS pre-sensitized monocytes/macrophages to secrete inflammatory cytokines in a TLR4-dependent manner (36, 45), we initially speculated that GzmA would affect DC functions in synergy with TLR4. However, instead, we found that GzmA stimulates pDCs in a TLR9 dependent manner as an adjuvant effect. We demonstrated that, after the internalization of GzmA, it becomes associated with mitochondria and endosomes in the cytoplasm and then colocalizes with TLR9 (-8 h). These results suggest that either endosomes or mitochondria, or both might be related to the activation of the TLR9 signaling pathway in pDC. Several studies have recently discussed the dynamic interaction between endosomes and mitochondria. By binding to mitochondria, iron and cholesterol were transferred from endosomes to mitochondria, thus enhancing mitochondrial metabolism (66–68). In addition, upon oxidative stress, the small GTPase Rab5, which can usually be found on early endosomes, was relocated to mitochondria for protecting cells (69). In our case, through this interaction, gzmA may traffic from endosome to mitochondria, where it might form complexes with mtDNA. Then, it is supposed that the GzmA-mtDNA complex may be engaged by TLR9, thus leading to activation of the downstream TLR9-MyD88 signaling pathway in pDCs. However, since this is our speculation, further studies will be required to define the precise mechanism.

Regarding the interesting difference between CpG-ODN and GzmA as TLR9 ligands, we found that FL-DCs as well as sorted pDCs secreted more IFN-α in response to CpG-ODN than to GzmA *in vitro*. In contrast, when we analyzed the differences in T cell responses by co-administrating with antigen, a better T cell response was seen with GzmA than with CpG-ODN. These findings indicate that the adjuvant activity of GzmA is better than that of CpG-ODN. The discrepancy between *in vitro* and *in vivo* activities could be explained by the instability of CpG-ODN *in vivo*. Because CpG-ODN is easily degraded by DNases (70), it is unstable and has a short half-life *in vivo* (70, 71).

In conclusion, GzmA is of interest not only because of its newly described adjuvant activity, but also its possible use in tumor therapy. The immunological findings presented here, including antigen-presentation, antigen-specific immunity and anti-tumor immunity, provide a new mechanism by which GzmA can link innate and adaptive immunity. In light of its biological properties, under different conditions, GzmA may promote different pathways, enhancing inflammation by stimulating TLR4 or TLR9 signaling or showing antitumor or antibacterial cytotoxicity via its protease activity. The current study of GzmA will be helpful in laying the groundwork for further basic studies of inflammation or tumor immunity and for establishment of novel vaccine strategies.

## Ethics Statement

This study was carried out in accordance with the recommendations of the Institutional Animal Care Committee at RIKEN.

## Author Contributions

KS and SF carried out all the mouse experiments, analyzed, and interpreted data. SY and KS performed microscopy observation and immunoprecipitation assay. MSa, NY, and MK supported the experiments. MI, CM-T, MK-N, and MS prepared the recombinant granzyme A and building its 3D structural model. KS and TW performed RNAseq. KS and SF designed, supervised most of the experiments and wrote the paper.

### Conflict of Interest Statement

The authors declare that the research was conducted in the absence of any commercial or financial relationships that could be construed as a potential conflict of interest. The reviewer MD and handling Editor declared their shared affiliation.
