# Supplementary material for: Granzyme A Stimulates pDCs to Promote Adaptive Immunity via Induction of Type I IFN
Source: Front Immunol. 2019 Jun 26;10:1450. doi: 10.3389/fimmu.2019.01450 (PMC6606709; doi:10.3389/fimmu.2019.01450)
Supplement: Supplementary file 1 [file Data_Sheet_1.PDF]

Supplementary Material

Granzyme A stimulates pDCs promoting adaptive immunity via induction of type I IFN

Kanako Shimizu[1], Satoru Yamasaki[1], Maki Sakurai[1], Noriko Yumoto[1], Mariko Ikeda[2], Chiemi Mishima-Tsumagari[2], Mutsuko Kukimoto-Niino[2], Takashi Watanabe[3], Masami Kawamura[1], Mikako Shirouzu[2] and Shin-ichiro Fujii[1, *]

Fig. S1-5

Supplementary Figure 1

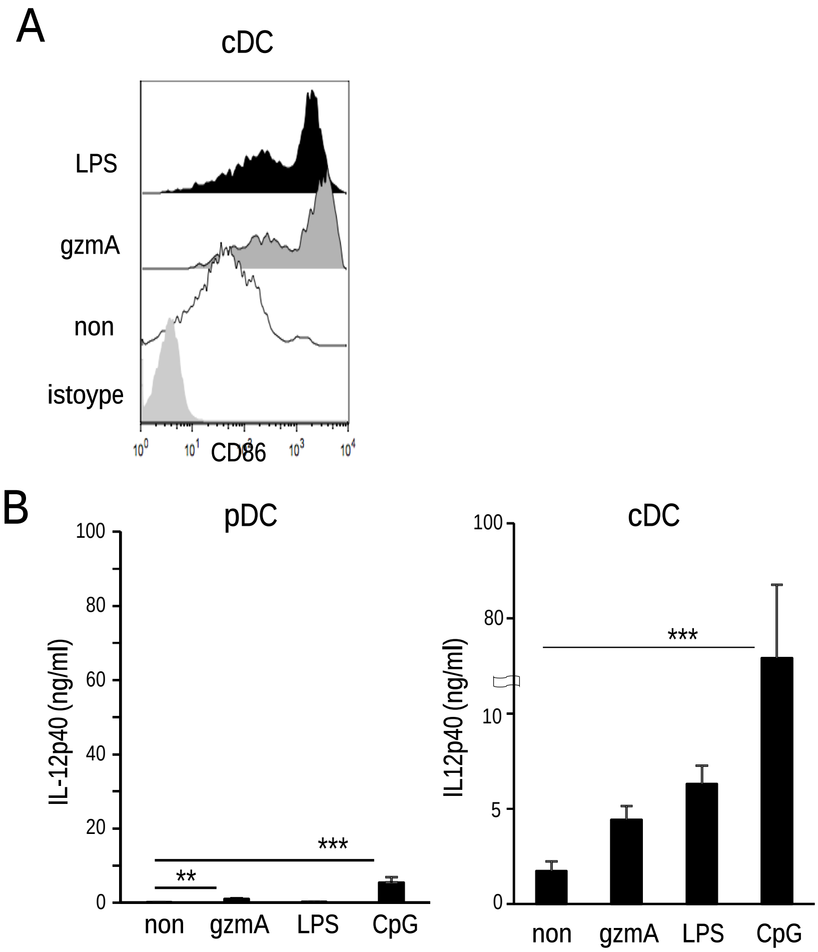

Fig. S1 GzmA promotes cDC maturation.
(A) Phenotypic maturation. FL-DCs were generated from BMMNCs from C57BL/6 mice in the presence of FLT3L. The expression of CD86 on cDCs ($CD11c^{+}B220^{-}$) among the FL-DCs was assessed 24 h after stimulation with gzmA (1 μg/ml), LPS (100 ng/ml) or nothing (non). (n=5) **(B)** Cytokine production. Sorted pDCs and cDCs from FL-DCs ($1x10^{5}$/well) were unstimulated (non) or stimulated with gzmA (1 μg/ml), LPS (100 ng/ml) or CpG (1 μg/ml). The supernatants were harvested 24 h later and assessed for IL-12p40 production by ELISA. (mean±SEM, n=4-6) ***p<0.01* (Kruskal-Wallis).

Supplementary Figure 2

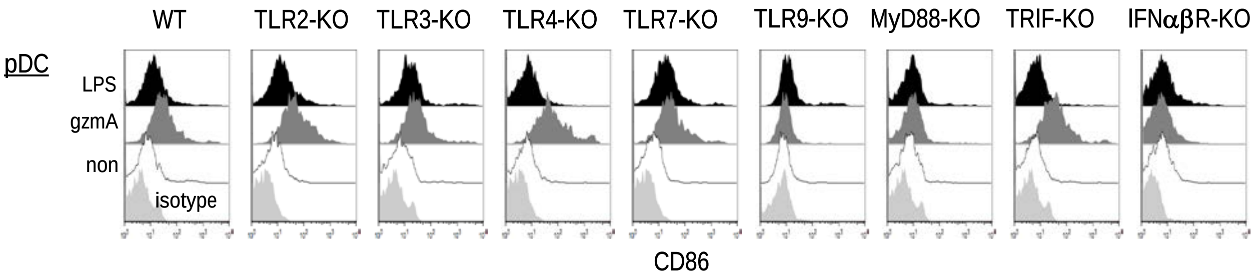

Fig. S2 Phenotypic maturation of pDCs stimulated with gzmA.
FL-DCs generated from WT or the indicated KO mice were stimulated with gzmA (1 μg/ml) or LPS (100 ng/ml). The expression of CD86 on pDCs ($CD11c^{lo}B220^{+}$) among FL-DCs was assessed 24 h later.

Supplementary Figure 3

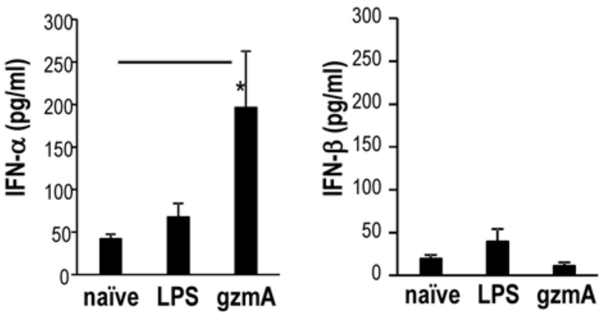

Fig. S3 Serum cytokines.
Serum cytokines. Mice were injected intravenously with gzmA (20 μg/mouse) or LPS (20 μ g/mouse). Sixteen hours later, serum was assayed for IFN-α and IFN-β (mean ±SEM, n=4).
*<*p*<0.05* (gzmA vs naive, Steel's Many-one Rank Sum test).

Supplementary Figure 4

A

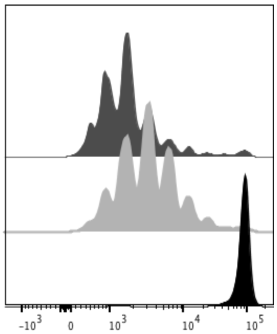

B

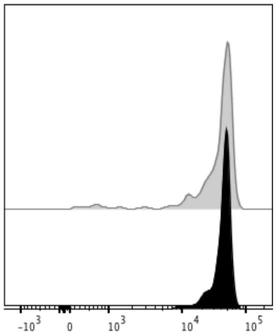

Fig. S4 OT-1 proliferation
Mice were administered with 1x10^6 CFSE-labeled OT-1 cells at day-1 and then immunized with cell-associated OVA (T/O) together with or without GzmA at day 0 (A).In some experiments, CFSE-OT-1 transferred mice were injected with or without GzmA at day 0 (B). Three days later, OT-1 proliferation by spleen cells was monitored by CFSE dilution. Data are representative of two independent experiments. (n=4).

Supplementary Figure 5

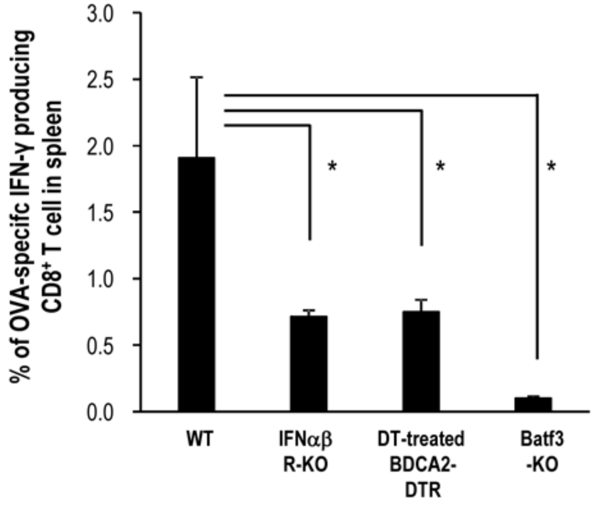

Fig. S5 Antigen specific $CD8^{+}$T cell response
As shown in Figure 7E, the immune response was measured in C57BL/6 mice and several KO mice immunized i.v. with T/O cells together with or without GzmA. In some experiments, BDCA2-DTR mice were treated with DT. IFN-g production by OVA-specific $CD8^{+}$ T cells in spleen were analyzed by flow cytometry on day 7.
All data are representative of two independent experiments with similar results (mean±SEM, n=4). *<p<0.05 (Steel's Many-one Rank Sum test)
